# Super-refractory status epilepticus and ketogenic diet in intensive care: a series report

**DOI:** 10.5935/0103-507X.20210089

**Published:** 2021

**Authors:** João Camões, Ana Hipólito Reis, Liliana Sousa, Ernestina Gomes

**Affiliations:** 1 Emergency and Intensive Care Department, Unidade Local de Saúde de Matosinhos - Matosinhos, Portugal.; 2 Nutrition Service, Unidade Local de Saúde de Matosinhos - Matosinhos, Portugal.

**Keywords:** Status epilepticus, Diet, ketogenic, Epilepsy, Critical care, Intensive care units

## Abstract

Super-refractory status epilepticus is defined as seizures that persist or reemerge in the setting of an intravenous anesthetic infusion for more than 24 hours. In recent years, attention has been driven to the potential benefits of a ketogenic diet in the management of these patients. However, the specific role of this strategy in the adult population, as well as its underlying mechanism of action and optimal time for the initiation and management of complications, remain widely debatable. We report a case series of three patients admitted to an intensive care unit due to super-refractory status epilepticus who were managed with a ketogenic diet and propose a clinical approach to its initiation, transition, and management of clinical intercurrences.

## INTRODUCTION

In the setting of neurologic emergencies, status epilepticus (defined as seizure or recurrent seizures lasting at least five minutes without return to baseline neurologic condition^(^1^)^ has a high prevalence and elevated morbimortality.

Refractory status epilepticus is defined as persistent seizures despite appropriate use of two intravenous medications, one of which is a benzodiazepine. Super-refractory status epilepticus (SRSE) is defined as seizures that persist or reemerge in the setting of an intravenous anesthetic infusion for more than 24 hours.^(^1^)^ Optimal management of patients in SRSE is controversial; however, the achievement of seizure control and return of baseline neurological function remains the goal of treatment.

In recent years, attention has been driven to the potential benefits of a ketogenic diet (KD) in the complex management of these patients.^(^1^-^4^)^ A ketogenic diet is a dietary regime focused on a reduction in carbohydrate intake alongside a relative increase in protein and fat to promote fat metabolism.^(^5^)^ Although the majority of the literature focuses on the pediatric population,^(^2^,^4^)^ there have been reports of its effectiveness in adults.^(^3^,^5^)^ Because of the paucity of evidence in the specific setting of SRSE, we present three cases of adult patients with SRSE treated with the addition of KD as adjunctive therapy in an intensive care unit (ICU) and suggest an approach to this specific population.

Informed written consent was obtained from all patients or legal representatives.

## CASES REPORT

### Case 1

A previously healthy 20-year-old male was admitted after isolated head trauma. An initial cerebral computed tomography (CT) scan showed hemorrhagic foci in the frontal parasagittal subcortical regions and right base ganglia. On the 7th day of hospital admission, the patient suffered a tonic-clonic seizure with no neurological recovery and was admitted to the ICU for 24 hours burst suppression (BS) cycle. Cerebral magnetic resonance imaging (MRI) showed axonal injury in both the temporal and frontal lobes, and central nervous system infection was excluded. After BS interruption, electroencephalogram (EEG) revealed poorly structured and slow-based brain electrogenesis with bilateral frontal delta rhythmic activity. The patient remained with EEG criteria of SRSE in the first 13 days of ICU hospitalization although being submitted to adequate BS cycles. On the 5th day of ICU admission, a fasting regimen aimed at inducing a ketose state was started. It was suspended after 36 hours due to several episodes of hypoglycemia. As we progressed with subsequent BS cycles with clinical recurrence of seizures, we reinitiated a fasting regimen, and ketonuria was achieved in 28 hours. A KD enteric formula composed of a 4:1 ratio (4g of fat to every 1g of protein) supplemented with minerals, essential fatty acids, and vitamins was then started, and a full dose was attained in 48 hours. Re-evaluation EEG after 68 hours of ketonuria showed no evidence of paroxistic activity. The patient maintained a KD with no further complications throughout the rest of hospitalization and was discharged 45 days later with no major neurological sequelae. The transition to a modified Atkins diet was initiated in the general ward, and subsequent increases in carbohydrate intake up to 45% of total energy were successfully tolerated. At the 3-month follow-up evaluation, the patient returned to his labor activity and was on an unrestricted diet, without clinical relapse of seizures.

### Case 2

A 38-year-old female developed a 3-day clinic of myalgia and fever, culminating in three episodes of tonic-clonic seizures and posterior admission to our emergency department in a stuporous state. The initial diagnostic workup included a normal CT scan and MRI, lumbar puncture with noninflammatory characteristics, a negative microbiological panel, normal blood inflammatory markers, and a negative neuronal antibody title panel. The therapeutic strategy in the first 24 hours included a BS cycle with electroencephalographic features of seizures after suspension of this strategy. The patient remained in nonconvulsive status epilepticus in each BS stop evaluation for 9 days. A fasting regimen was then started to promote a ketosis state. However, after 4 days of this strategy, ketonuria was still absent, and we introduced a KD with a 4:1 ratio supplemented with minerals and vitamins titrated to the target dose within 10 days. An electroencephalogram performed after 48 hours of KD initiation showed no paroxysmal activity, and ketonuria was achieved 8 days after initiation of this strategy.

On the 14^th^ day of enteric feeding, major gastric stasis and diarrhea developed. After switching to total parenteral nutrition and manipulating to a ketogenic profile, the patient gradually improved, and a transition to a modified Atkins diet was possible in the fourth week of hospitalization. Carbohydrates were progressively introduced, and she was discharged with a nutritional plan for carbohydrate dose progression. At the follow-up consultation, she was stable and had returned to a liberal diet without recurrence of symptoms.

### Case 3

A 20-year-old female with no known medical problems was admitted to our emergency department after a 4-day course of fever, vomiting, and diarrhea. At presentation, she had a tonic-clonic seizure with no neurological recovery, leading to intubation and ICU admission. The initial diagnostic workup included normal cerebral CT scan and MRI. Lumbar puncture was negative for infection. We induced 4 BS cycles with EEG criteria of seizures after each pause of sedation. We then started a fasting regimen to achieve a ketosis state. Although ketonuria was absent, a KD of a 4:1 ratio was initiated after 48 hours. Progression to full dose was not achieved due to severe ileus, which led to exchange to a parenteral formula on the 12th day of hospitalization. The patient remained with seizure EEG criteria in subsequent evaluations. Clinical deterioration occurred on the 19th day of ICU admission, with severe hypertriglyceridemia and de novo septic shock, leading to suspension of de parenteral nutrition. The patient evolved refractory shock and died on the 21st day of hospitalization.

**Figure 1:**
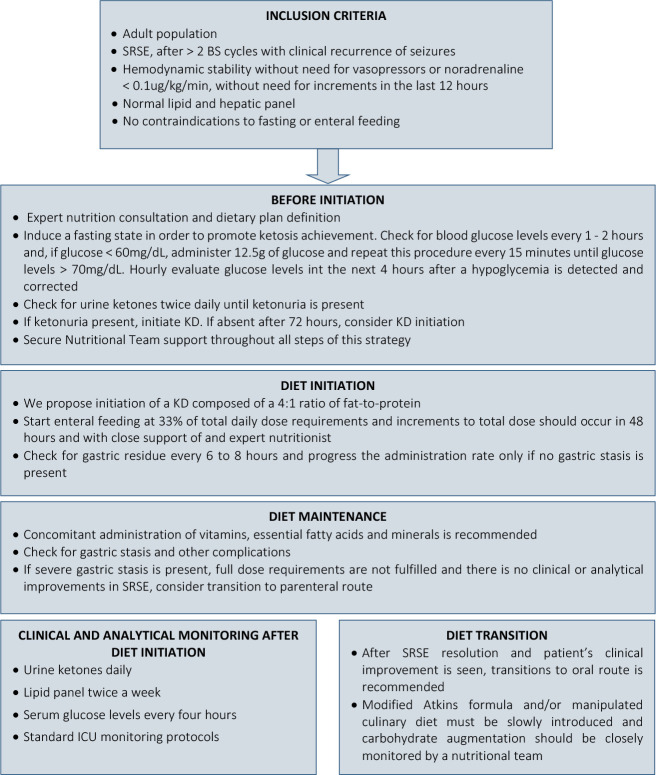
Clinical approach algorithm.

The clinical evolution of all cases is summarized in table 1. Due to lack of availability in the literature, we propose a clinical approach algorithm to patients in SRSE in whom KD may be considered (Figure 1). Several considerations regarding diet initiation, maintenance, and transition, as well as laboratory and clinical monitoring, were considered.

**Table 1:** Clinical evolution of all cases

Case	Age (years)	Gender	NUTRIC Score at admission (points)	SRSE duration (days)	SRSE duration after KD initiation (days)	KD duration in ICU (days)	Fasting time (hours)	Days until ketonuria achievement after fasting	Major intercurrences	Outcome
1	20	Male	2	13	3	32	1^st^: 36 2^nd^: 28	1	Hypoglycemia	Alive
2	38	Female	4	15	2	41	80	N/A	Gastric stasis Hypoglycemia	Alive
3	20	Female	2	20	11	11	48	N/A	Hypertriglyceridemia Ileus Septic shock	Death

ICU - intensive care unit; KD - ketogenic diet; NUTRIC - Nutrition Risk in the Critically Ill. SRSE - super-refractory status epilepticus; N/A - not achieved.

SRSE - super-refractory status epilepticus BS - burst suppression; KD - ketogenic diet; ICU - intensive care unit.

## DISCUSSION

A KD has been used as an adjunctive therapy for infants with refractory epilepsy since 1920 and, in recent years, for adults.^(^6^)^ However, in contrast with evidence regarding seizure management in children,^(^2^)^ its promising use in adults has only recently been studied.^(^1^,^3^,^6^,^7^)^ To our knowledge, the only prospective study in this field^(^7^)^ reported a 73% resolution of SRSE within 1 week of KD initiation.

The underlying mechanisms of action of KD are still largely unknown.^(^3^,^4^)^ It has been postulated that the presence of acetoacetate could confer some grade of seizure control; however, there is an inconsistent correlation between ketosis and seizure control.^(^8^)^ Ketosis can slow energy production and potentiate GABA inhibition. Several other nonketosis-related mechanisms have been hypothetically linked to confer some grade of seizure control.^(^4^)^

Another point of discussion is the definition of ketosis presence, but most reports agree that the presence of ketonuria is indicative of a ketosis state. Since ketosis achievement before initiation of supplementary diet is debatable, the optimal time for initiation of KD is unknown.^(^3^,^6^,^7^)^

We introduced KD (with carbohydrate restriction to 1.5% of the total caloric value) after exclusion of surgically treatable causes and metabolic corrections were achieved. Since there are several side effects of KD, we proposed initiating KD after the 3rd or 4th BS cycles, since some patients respond better to other antiepileptic iv drugs.

In our practice, our primary objective was the achievement of a ketosis state before introducing a ketogenic formula. However, in two patients, due to recurrent hypoglycemia and the need for various glycosylated fluids to surpass this problem (and thus prevent ketone production), we started ketogenic dietary supplementation before the achievement of ketonuria, with apparent clinical benefit. Since the underlying mechanism of action of KD is widely debatable and reports in the literature are contradictory, our position was to wait for ketonuria to be present if no clinical harm was seen with this strategy.^(^1^)^

Multiple ketogenic diets are available (Classic KD, Modified Atkins Diet - MAD, Medium Chain Triglyceride - MCT Diet, Low Glycemic Index - LGI).^(^4^)^ Classic KD is composed of a 4 to 1 proportion of fat to nonketogenic proteins and carbohydrates and is the most common KD reported in the literature.

A MCT ketogenic diet has more ketogenic potential than classic CD since MCT is more readily absorbed than long-chain triglycerides.^(^4^)^ A recent report^(^3^)^ used an MCT diet in an adult case of SRSE with clinical success and a low rate of complications, suggesting the potential benefit of this diet in patients with SRSE who cannot tolerate classic KD due to hypertriglyceridemia.

The MAD is a form of ketogenic diet (typically restricted to 10 - 20g of carbohydrates per day) where fat components account for approximately 65% of total calories.^(^4^)^ Since it is more liberalized, it is a treatment option in ambulatory patients or in those who are transitioning to a less restrictive therapy. For LGI, an even more liberal dietetic approach is used, with apparent clinical benefits in pediatric populations.

Our clinical approach was to initiate KD in a classical formula and, after clinical improvement, slowly transition to MAD and LGI. Two of our patients were observed at follow-up consultation 3 months after hospital discharge. A nutrition plan and consultation were strictly followed during this period, and both progressed to a liberalized diet with no clinical recurrence of seizures.

As previously reported,^(^1^)^ hypoglycemia is a common complication in patients with KD. We defined a minimum glucose level of 60mg/dL for intervention. One of our patients had various episodes of severe hypoglycemia during the fasting period that ultimately led to a postponed introduction of KD. During KD feeding, however, we had no reported episodes of blood glucose levels less than 60mg/dL. As such, we propose to monitor blood glucose levels every 2 hours and after KD initiation to closely monitor glucose levels every 4 hours.

We monitored triglyceride levels twice a week since lipid derangement is one of the most common side effects of KD.^(^4^)^ One of our cases developed severe hypertriglyceridemia under KD. However, this patient was under parenteral nutrition, and this complication was more likely associated with the parenteral route of administration than with the ketogenic profile of the diet. As with conventional parenteral nutrition concerns, it has been established that previous triglyceride (TG) levels, body mass index and high carbohydrate concentrations (> 3.1g/kg/day) are risk factors for the development of hypertriglyceridemia.^(^9^)^ Since our KD formula had a low carbohydrate concentration, we assumed that variables other than the specific composition of our KD were responsible for the elevation of TG levels.

To our knowledge, the majority of KD administrations in adults are by the enteral route. The feasibility and safety of parenteral KD in adults has yet to be assessed. There are reports of parenteral KD in pediatric populations^(^10^)^ with some interesting results, but there are very limited reports of its feasibility and safety in adult populations and, to our knowledge, none in the presence of SRSE. We decided to initiate parenteral KD in two of our cases due to severe gastric stasis, a common feature during KD.^(^4^,^5^)^ Clinical improvement was seen in both cases; however, in one case, this strategy was abandoned because of de *novo* septic shock.

Regarding nondietary complications, we reported severe hypocalcemia in two patients treated with phenytoin that led to its discontinuation. Hypocalcemia can deteriorate seizure control due to vitamin D deficiency, and close monitoring is required. We opted to supplement our patients with mineral, vitamin, and calcium supplements, mainly those that are not synthesized by the human body.

## CONCLUSION

In conclusion, a ketogenic diet may play a role in the treatment of super-refractory status epilepticus.
